# Supplementary material for: Bacterial persistence is an active σS stress response to metabolic flux limitation
Source: Mol Syst Biol. 2016 Sep 21;12(9):882. doi: 10.15252/msb.20166998 (PMC5043093; doi:10.15252/msb.20166998)
Supplement: Supplementary file 1 — Appendix [file MSB-12-882-s005.docx]

**Appendix**

Bacterial persistence is an active σ^S^ stress response to metabolic flux limitation

Jakub Leszek Radzikowski^1^, Silke Vedelaar^1^, David Siegel^3^, Alvaro Dario Ortega^1^, Alexander Schmidt^2^, Matthias Heinemann^1*^

^1^Molecular Systems Biology, Groningen Biomolecular Sciences and Biotechnology Institute, University of Groningen, Nijenborgh 4, 9747 AG Groningen, The Netherlands

^2^Biozentrum, University of Basel, Klingelbergstrasse 50/70, 4056 Basel, Switzerland

^3^Analytical Biochemistry Group, Groningen Research Institute of Pharmacy, University of Groningen, Antonius-Deusinglaan 1, 9713 AV Groningen, The Netherlands

^*^Corresponding author: m.heinemann@rug.nl (phone +31 50 363 8146)

# Table of Contents

[Appendix Figures 3](#_Toc459401679)

[Appendix Figure S1 – Estimation of the effect of the small fraction of growing cells after a nutrient switch on the determined persister phenotype. 3](#_Toc459401680)

[Appendix Figure S2 - Heat map of expression levels of proteins involved in *E. coli* central metabolic pathways. 5](#_Toc459401681)

[Appendix Tables 6](#_Toc459401682)

[Appendix Table S1 – *E. coli* cell size on glucose, fumarate, as well as during entry into persistence and entry into starvation 6](#_Toc459401683)

[Appendix Table S2 – Physiological parameters of cells growing exponentially on fumarate, and in cells 8 hours after entry into persistence or starvation 7](#_Toc459401684)

[Appendix Table S3 – GOterms differentiating growing and persister cells, or starved and persister cells. 8](#_Toc459401685)

[Appendix Table S4 – Fold change in concentrations of sigma factors 10](#_Toc459401686)

[Appendix Table S5 – Fold change in concentration of σ^S^ regulon proteins with more than 2-fold change at 8 hours in persister cells. 11](#_Toc459401687)

[Appendix Table S6 – Change in antibiotic tolerance to ampicillin after nutrient-shift in mutant strains. 13](#_Toc459401688)

[Appendix Table S7 – Primers used for TAS transcript quantification 14](#_Toc459401689)

[Appendix Table S8 – Settings of STEM software 15](#_Toc459401690)

[Appendix Texts 16](#_Toc459401691)

[Appendix Text S1: Evaluation of the effects of the presence of non-persister cells on the determined persister phenotype 16](#_Toc459401692)

[Appendix Reference List 18](#_Toc459401693)

# Appendix Figures

## Appendix Figure S1 – Estimation of the effect of the small fraction of growing cells after a nutrient switch on the determined persister phenotype.

**
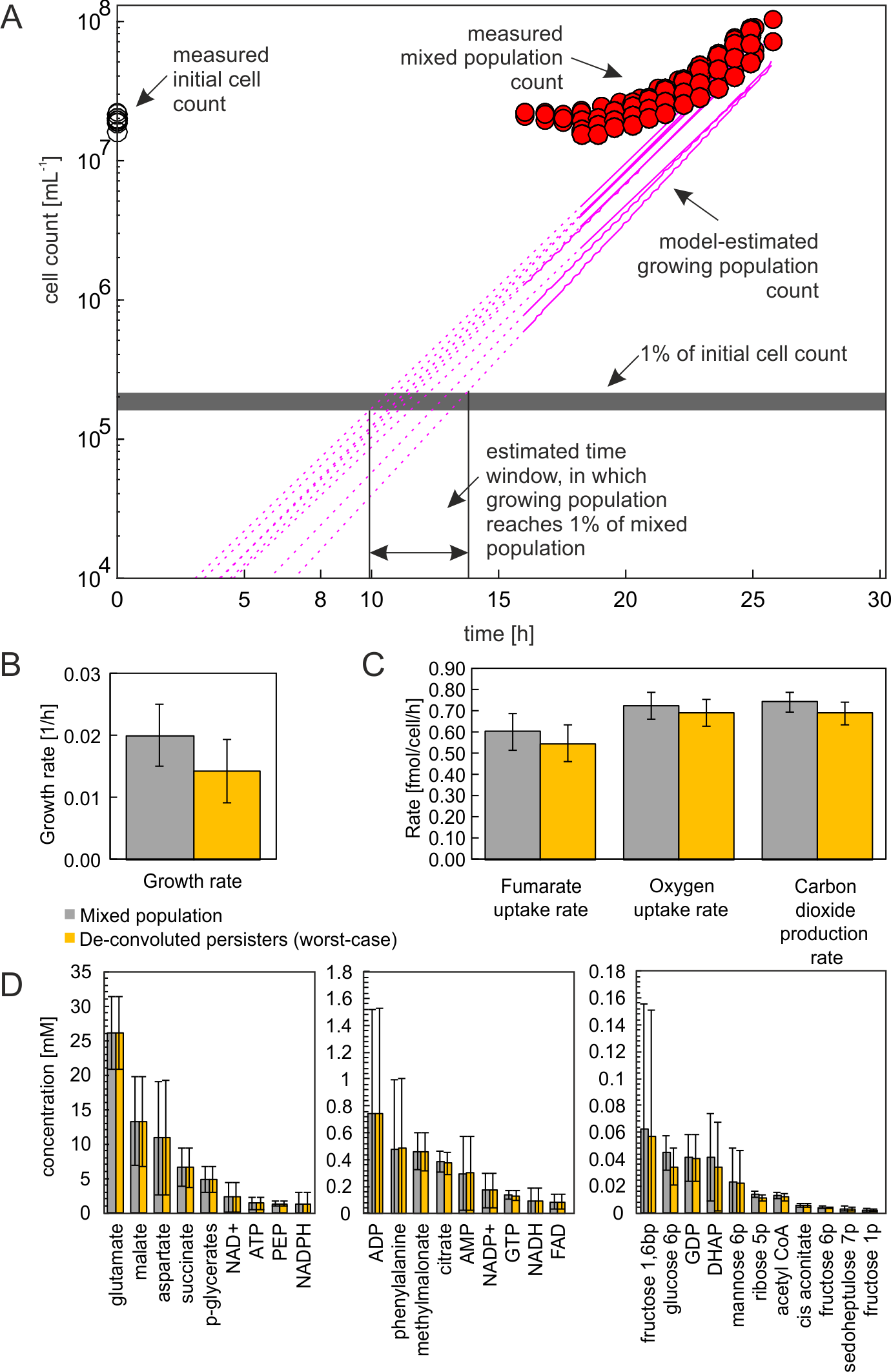
**

**(A)** Cell count measurements at multiple time points after the switch from glucose to 2 g L^-1^ fumarate, from 9 independent experiments. In these experiments, cells were stained with a fluorescent dye as described before (Kotte et al., 2014). Non-/slow-growing cells retain fluorescence, while it is halved in growing cells with each division, resulting in a bimodal distribution of fluorescence intensities in a population. The violet lines represent model fits characterizing the number of growing cells as performed before (Kotte et al., 2014). Specifically, the model fits a bi-Gaussian distribution to the dynamic fluorescence and the cell count data, and estimates the sizes of the sub-populations. Open circles – initial cell density, red circles – total cell count, violet line – growing cell fit (solid) or extrapolation of the fit (dashed). The grey bar represents 1% of the initial cell density. The intersects of the grey bar and the extrapolated growing cell fits show that the growing population reached 1% of the initial cell density approximately 10 to 15 hours after the switch. The estimated fraction of growing cells in the total measured population, in combination with the phenotype measurements of mixed population and the measurements of the phenotype of cells growing normally on fumarate were used to estimate the effect of the growing cells present in the population (cf. Appendix Text S1). Panels **(B-D)** show a comparison of the mixed population measurements (grey bars) and the de-convoluted worst-case scenario (yellow bars) of **(B)** the growth rates, **(C)** the physiological rates, and **(D)** the metabolite concentrations. Error bars indicate 95% confidence intervals of the mean. Data from replicate experiments.

## Appendix Figure S2 - Heat map of expression levels of proteins involved in *E. coli* central metabolic pathways.


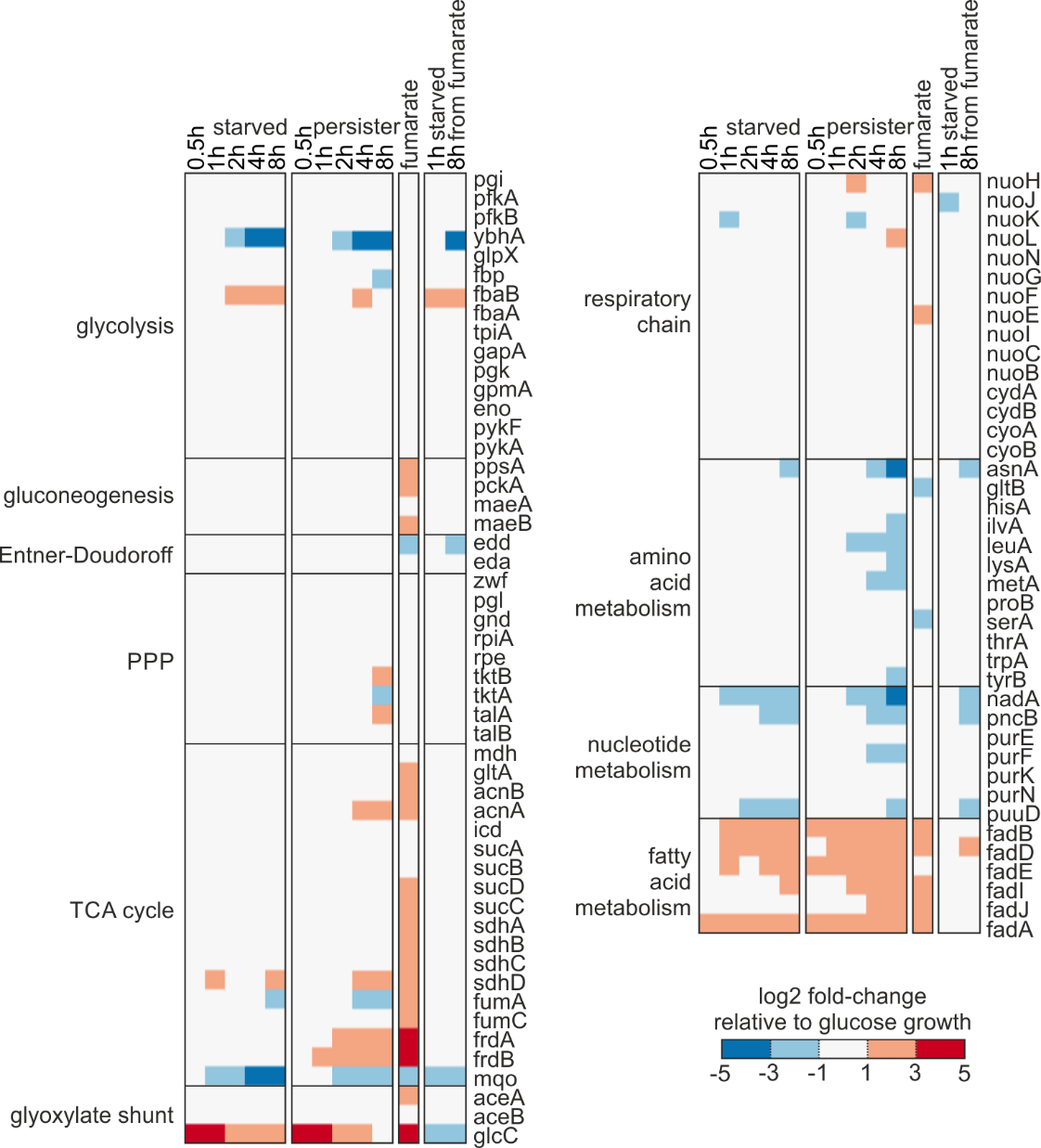


The data show log2-fold change of protein levels (relative to cells growing on glucose) in starved cells, persister cells, cells growing on fumarate and log2-fold change (relative to cells growing on fumarate) in cells starved from fumarate (in relation to fumarate). Gene lists for pathways were generated based on data from EcoCyc (Keseler et al., 2013). All genes with protein expression data available shown. White color indicates less than 2-fold change (i.e. log2-fold change between -1 and 1) in expression.

# Appendix Tables

## Appendix Table S1 – *E. coli* cell size on glucose, fumarate, as well as during entry into persistence and entry into starvation

| condition [cell number] | mean volume [fL] | sd | mean width [um] | sd | mean length [um] | sd |
| --- | --- | --- | --- | --- | --- | --- |
| fumarate [n=116] | 1.11 | 0.58 | 0.89 | 0.18 | 2.45 | 0.55 |
| glucose [n=104] | 2.15 | 0.84 | 1.15 | 0.18 | 2.87 | 0.56 |
| persister 0.5h [n=143] | 0.89 | 0.34 | 0.88 | 0.12 | 2.11 | 0.55 |
| persister 1h [n=154] | 0.73 | 0.34 | 0.83 | 0.14 | 1.91 | 0.41 |
| persister 2h [n=158] | 0.55 | 0.26 | 0.76 | 0.14 | 1.72 | 0.40 |
| persister 4h [n=183] | 0.60 | 0.25 | 0.81 | 0.12 | 1.70 | 0.39 |
| persister 8h [n=171] | 0.61 | 0.26 | 0.81 | 0.13 | 1.73 | 0.44 |
| starved 0.5h [n=138] | 1.00 | 0.43 | 0.94 | 0.17 | 2.09 | 0.52 |
| starved 1h [n=176] | 0.71 | 0.45 | 0.85 | 0.17 | 1.73 | 0.51 |
| starved 2h [n=155] | 0.63 | 0.33 | 0.78 | 0.13 | 1.88 | 0.49 |
| starved 4h [n=124] | 0.75 | 0.39 | 0.86 | 0.16 | 1.80 | 0.45 |
| starved 8h [n=172] | 0.92 | 0.40 | 0.95 | 0.15 | 1.95 | 0.41 |

Cell volumes, widths and lengths in different conditions and time points measured with super-resolution microscopy. Number of analyzed cells (n) is indicated for each condition. The volumes were used for the determination of the intracellular metabolite concentrations.

## Appendix Table S2 – Physiological parameters of cells growing exponentially on fumarate, and in cells 8 hours after entry into persistence or starvation

|  | **Cells growing on fumarate (2 g L^-1^)** | **Persister cells** | **Starved cells** |
| --- | --- | --- | --- |
| **Growth rate**  **[h^-1^]** | 0.56 ± 0.08 | 0.02 ± 0.005 | -0.005 ± 0.005 |
| **Fumarate uptake rate**  **[fmol cell^-1^ h^-1^]** | 5.47 ± 1.28 | 0.60 ± 0.08 | N/A |
| **Oxygen uptake rate**  **[fmol cell^-1^ h^-1^]** | 3.07 ± 1.19 | 0.72 ± 0.05 | N/A |
| **Carbon dioxide production rate**  **[fmol cell^-1^ h^-1^]** | 5.08 ± 1.77 | 0.74 ± 0.06 | N/A |

Physiological parameters of cells growing on 2 g L^-1^ fumarate, persister cells in fumarate medium 8 hours after the nutrient shift, and starved cells. Mean values and 95% confidence intervals of the mean shown.

## Appendix Table S3 – GOterms differentiating growing and persister cells, or starved and persister cells.

| PCA of persister, glucose-growing and fumarate-growing cells’ proteomes | | | | | |
| --- | --- | --- | --- | --- | --- |
| GOterms characterizing growing phenotypes, based on dimension 1 | | | | | |
| Rank | GO ID | | GOterm | | |
| 1 | GO:0009239 | | enterobactin biosynthetic process | | |
| 2 | GO:0006790 | | sulfur compound metabolic process | | |
| 3 | GO:0009432 | | SOS response | | |
| 4 | GO:0009435 | | NAD biosynthetic process | | |
| 5 | GO:0009086 | | methionine biosynthetic process | | |
| 6 | GO:0046656 | | folic acid biosynthetic process | | |
| 7 | GO:0006310 | | DNA recombination | | |
| 8 | GO:0006261 | | DNA-dependent DNA replication | | |
| 9 | GO:0042398 | | cellular modified amino acid biosynthetic process | | |
| 10 | GO:0009226 | | nucleotide-sugar biosynthetic process | | |
| GOterms characterizing persister phenotype, based on dimension 1 | | | | | |
| Rank | GO ID | | GOterm | | |
| 1 | GO:0006096 | | glycolytic process | | |
| 2 | GO:0006970 | | response to osmotic stress | | |
| 3 | GO:0044260 | | cellular macromolecule metabolic process | | |
| 4 | GO:0006457 | | protein folding | | |
| 5 | GO:0042594 | | response to starvation | | |
| 6 | GO:0006006 | | glucose metabolic process | | |
| 7 | GO:0006401 | | RNA catabolic process | | |
| 8 | GO:0009063 | | cellular amino acid catabolic process | | |
| 9 | GO:0006281 | | DNA repair | | |
| 10 | | GO:0009252 | | peptidoglycan biosynthetic process | |
| PCA of persister, fumarate-starved and glucose-starved cells’ proteomes | | | | | |
| GOterms characterizing starved phenotypes, based on dimension 1 | | | | | |
| Rank | | GO ID | | | GOterm |
| 1 | | GO:0006099 | | | tricarboxylic acid cycle |
| 2 | | GO:0009152 | | | purine ribonucleotide biosynthetic process |
| 3 | | GO:0046129 | | | purine ribonucleoside biosynthetic process |
| 4 | | GO:0009435 | | | NAD biosynthetic process |
| 5 | | GO:0072528 | | | pyrimidine-containing compound biosynthetic process |
| 6 | | GO:0006261 | | | DNA-dependent DNA replication |
| 7 | | GO:0046487 | | | glyoxylate metabolic process |
| 8 | | GO:0015949 | | | nucleobase-containing small molecule interconversion |
| 9 | | GO:0009432 | | | SOS response |
| 10 | | GO:0006732 | | | coenzyme metabolic process |
| GOterms characterizing persister phenotype, based on dimension 1 | | | | | |
| Rank | | GO ID | | | GOterm |
| 1 | | GO:0006970 | | | response to osmotic stress |
| 2 | | GO:0009252 | | | peptidoglycan biosynthetic process |
| 3 | | GO:0006412 | | | translation |
| 4 | | GO:0006096 | | | glycolytic process |
| 5 | | GO:0006401 | | | RNA catabolic process |
| 6 | | GO:0006281 | | | DNA repair |
| 7 | | GO:0006006 | | | glucose metabolic process |
| 8 | | GO:0042493 | | | response to drug |
| 9 | | GO:0044260 | | | cellular macromolecule metabolic process |
| 10 | | GO:0044267 | | | cellular protein metabolic process |

Ranked lists of 10 most enriched GOterms revealed after PCA and GOterm enrichment analyses of proteome data of persister cells, cells growing on glucose and cells growing on fumarate; as well as proteome data of persister cells, starved cells switched from glucose and starved cells switched from fumarate.

## Appendix Table S4 – Fold change in concentrations of sigma factors

|  | entry into persistence from glucose | | | | | entry into starvation from glucose | | | | | growth on fumarate | entry into starvation from fumarate | |
| --- | --- | --- | --- | --- | --- | --- | --- | --- | --- | --- | --- | --- | --- |
| gene | **0.5h** | **1h** | **2h** | **4h** | **8h** | **0.5h** | **1h** | **2h** | **4h** | **8h** |  | **1h** | **8h** |
| rpoD | **1.07** | **1.02** | **1.11** | **1.37** | **1.54** | **1.03** | **1.08** | **1.14** | **1.15** | **1.34** | **0.88** | **1.02** | **1.26** |
| rpoE | **1.04** | **1.06** | **1.14** | **1.39** | **1.50** | **1.14** | **1.17** | **1.10** | **1.18** | **1.40** | **0.78** | **0.88** | **1.05** |
| rpoN | **0.86** | **0.86** | **0.86** | **0.87** | **1.00** | **0.94** | **0.87** | **0.90** | **0.86** | **0.85** | **0.99** | **0.89** | **0.90** |
| rpoS | **5.45** | **4.28** | **3.83** | **3.34** | **3.05** | **3.91** | **3.49** | **2.54** | **2.04** | **2.01** | **0.71** | **4.09** | **2.21** |
| rpoZ | **0.86** | **0.94** | **0.88** | **1.12** | **1.31** | **0.93** | **0.91** | **0.85** | **1.04** | **0.94** | **1.01** | **0.83** | **0.89** |

Fold change in the concentrations of sigma factors in cells during the entry into persistence and starvation, and during growth on fumarate, compared to cells growing on glucose. During entry into persistence from glucose, and entry into starvation from either glucose or fumarate, sigma factor S is more than 2-fold more abundant than during growth on glucose.

## Appendix Table S5 – Fold change in concentration of σ^S^ regulon proteins with more than 2-fold change at 8 hours in persister cells.

|  | entry into persistence | | | | | entry into starvation | | | | |
| --- | --- | --- | --- | --- | --- | --- | --- | --- | --- | --- |
| gene | 0.5h | 1h | 2h | 4h | 8h | 0.5h | 1h | 2h | 4h | 8h |
| acnA | 1.11 | 1.45 | 1.96 | 2.86 | 3.29 | 0.96 | 1.15 | 1.29 | 1.45 | 1.53 |
| acs | 2.25 | 3.51 | 5.08 | 6.58 | 6.39 | 1.78 | 2.51 | 3.01 | 2.95 | 3.03 |
| actP | 1.82 | 2.75 | 3.68 | 4.87 | 5.29 | 1.42 | 1.99 | 2.14 | 1.83 | 1.73 |
| aidB | 0.96 | 1.72 | 3.48 | 6.03 | 6.95 | 1.73 | 1.76 | 2.74 | 3.14 | 3.32 |
| aldB | 2.80 | 5.24 | 10.08 | 17.15 | 26.34 | 2.27 | 4.36 | 5.47 | 6.24 | 7.04 |
| araF | 2.89 | 4.26 | 6.36 | 14.12 | 19.20 | 2.16 | 3.66 | 6.27 | 6.74 | 8.56 |
| araG | 0.23 | 0.56 | 2.05 | 2.78 | 11.08 | 0.37 | 0.21 | 2.25 | 1.22 | 3.53 |
| astA | 1.56 | 3.71 | 6.97 | 13.84 | 13.70 | 1.81 | 2.82 | 5.67 | 5.64 | 6.73 |
| astB | 0.38 | 0.68 | 1.76 | 2.67 | 2.37 | 0.48 | 0.43 | 1.08 | 0.87 | 0.83 |
| astC | 2.05 | 3.51 | 5.82 | 8.07 | 5.48 | 2.11 | 3.16 | 4.47 | 4.83 | 4.56 |
| astD | 1.66 | 1.97 | 3.51 | 7.01 | 4.96 | 1.77 | 2.14 | 2.42 | 3.49 | 4.64 |
| astE | 0.81 | 5.86 | 14.61 | 44.08 | 42.93 | 1.54 | 4.88 | 12.85 | 13.67 | 8.03 |
| bolA | 3.13 | 4.03 | 5.49 | 4.52 | 4.18 | 3.17 | 3.96 | 4.74 | 4.66 | 3.47 |
| cbpA | 0.94 | 1.16 | 1.29 | 1.75 | 2.09 | 0.87 | 0.88 | 0.96 | 1.12 | 1.17 |
| cfa | 0.17 | 0.14 | 0.08 | 0.13 | 0.20 | 0.11 | 0.03 | 0.00 | 0.01 | 0.01 |
| csiD | 18.01 | 16.75 | 12.98 | 12.89 | 15.03 | 17.68 | 19.31 | 18.96 | 9.07 | 8.19 |
| csiE | 4.56 | 9.22 | 17.30 | 18.46 | 20.04 | 3.69 | 6.66 | 9.11 | 7.65 | 8.71 |
| ddpA | 1.11 | 1.42 | 1.57 | 2.60 | 3.22 | 1.16 | 1.30 | 1.23 | 1.28 | 1.11 |
| dps | 1.01 | 1.43 | 2.17 | 1.98 | 2.45 | 0.89 | 1.59 | 2.17 | 2.16 | 2.32 |
| ecnB | 1.42 | 1.98 | 2.61 | 4.16 | 5.71 | 1.32 | 1.60 | 2.06 | 2.16 | 2.34 |
| fadL | 25.56 | 30.61 | 58.85 | 36.89 | 19.40 | 19.99 | 15.70 | 10.74 | 7.26 | 6.66 |
| fic | 1.35 | 2.03 | 2.44 | 3.75 | 4.82 | 1.38 | 1.74 | 2.05 | 2.28 | 2.66 |
| frdA | 1.64 | 1.82 | 2.88 | 3.78 | 4.54 | 1.13 | 1.41 | 1.80 | 1.45 | 1.53 |
| frdB | 1.95 | 2.39 | 3.05 | 4.04 | 4.57 | 1.29 | 1.38 | 1.30 | 1.53 | 1.78 |
| ftsB | 0.88 | 0.89 | 0.70 | 0.56 | 0.37 | 1.05 | 0.79 | 0.58 | 0.45 | 0.27 |
| gadA | 0.25 | 0.60 | 1.60 | 4.44 | 18.24 | 0.30 | 0.25 | 0.90 | 1.48 | 1.86 |
| gadB | 1.10 | 1.49 | 2.13 | 5.78 | 13.51 | 1.09 | 1.11 | 1.58 | 2.07 | 1.28 |
| glgS | 4.74 | 11.69 | 24.69 | 36.15 | 48.07 | 5.77 | 9.84 | 14.77 | 27.57 | 25.35 |
| gmr | 4.73 | 4.44 | 8.02 | 9.59 | 4.81 | 3.35 | 5.00 | 5.50 | 1.91 | 1.70 |
| hchA | 1.59 | 2.59 | 5.21 | 7.60 | 11.70 | 1.37 | 1.88 | 2.96 | 2.84 | 3.51 |
| hdeA | 0.56 | 0.67 | 0.78 | 2.41 | 5.48 | 0.39 | 0.45 | 0.60 | 0.76 | 0.81 |
| hdeB | 0.61 | 0.80 | 0.99 | 4.55 | 9.92 | 0.41 | 0.46 | 0.82 | 0.97 | 1.16 |
| hmp | 1.03 | 0.92 | 0.83 | 0.35 | 0.36 | 1.08 | 0.99 | 0.83 | 0.70 | 0.58 |
| ihfA | 1.20 | 1.38 | 1.62 | 2.08 | 2.31 | 1.14 | 1.47 | 1.29 | 1.25 | 1.33 |
| ihfB | 1.22 | 1.33 | 1.50 | 2.10 | 2.37 | 1.23 | 1.27 | 1.32 | 1.30 | 1.53 |
| katE | 0.37 | 0.27 | 0.17 | 0.12 | 0.16 | 0.30 | 0.24 | 0.16 | 0.10 | 0.11 |
| ldcC | 1.56 | 1.60 | 2.14 | 2.61 | 3.29 | 0.94 | 1.15 | 1.36 | 1.53 | 2.19 |
| lhgO | 1.37 | 1.96 | 1.90 | 2.20 | 3.78 | 1.34 | 1.64 | 1.73 | 2.01 | 2.34 |
| lsrA | 1.05 | 2.60 | 2.95 | 2.74 | 3.74 | 1.54 | 1.97 | 1.36 | 1.58 | 2.08 |
| lsrB | 1.60 | 5.07 | 5.02 | 7.41 | 9.05 | 1.24 | 2.91 | 3.70 | 4.37 | 3.98 |
| lsrF | 1.49 | 4.28 | 5.45 | 6.06 | 5.69 | 1.29 | 2.87 | 4.02 | 3.65 | 2.71 |
| lsrG | 2.28 | 5.75 | 5.53 | 4.95 | 7.90 | 1.29 | 3.58 | 4.76 | 3.05 | 2.99 |
| mdtA | 1.63 | 1.80 | 2.09 | 2.40 | 2.19 | 1.62 | 1.95 | 1.50 | 1.92 | 2.32 |
| mglA | 1.42 | 1.54 | 2.04 | 2.16 | 2.42 | 1.52 | 1.61 | 1.80 | 1.57 | 1.62 |
| mglC | 3.03 | 3.23 | 3.50 | 4.10 | 7.79 | 2.99 | 3.27 | 4.24 | 3.08 | 3.21 |
| msyB | 1.26 | 1.84 | 2.24 | 3.80 | 4.91 | 1.22 | 1.30 | 1.63 | 1.84 | 1.96 |
| osmB | 1.25 | 2.77 | 4.67 | 8.22 | 8.42 | 1.35 | 2.33 | 2.88 | 4.32 | 3.40 |
| osmC | 1.45 | 1.71 | 1.77 | 2.69 | 3.57 | 1.66 | 1.63 | 1.78 | 1.86 | 2.01 |
| osmE | 1.30 | 1.65 | 2.02 | 2.94 | 3.46 | 1.07 | 1.16 | 1.36 | 1.33 | 1.40 |
| osmY | 1.17 | 1.13 | 0.83 | 1.91 | 2.86 | 1.06 | 0.95 | 1.24 | 1.02 | 0.94 |
| otsA | 1.06 | 1.19 | 1.37 | 1.61 | 2.57 | 0.96 | 1.06 | 1.04 | 0.96 | 1.17 |
| poxB | 1.03 | 1.18 | 1.43 | 1.78 | 2.34 | 0.98 | 0.98 | 1.12 | 1.11 | 1.15 |
| puuA | 0.41 | 0.25 | 0.09 | 0.09 | 0.15 | 0.39 | 0.30 | 0.05 | 0.20 | 0.22 |
| puuB | 0.13 | 0.05 | 0.02 | 0.06 | 0.05 | 0.09 | 0.14 | 0.05 | 0.03 | 0.04 |
| puuC | 0.57 | 0.51 | 0.34 | 0.15 | 0.11 | 0.59 | 0.49 | 0.34 | 0.21 | 0.18 |
| puuD | 0.90 | 0.92 | 0.86 | 0.66 | 0.18 | 0.70 | 0.69 | 0.49 | 0.33 | 0.16 |
| puuE | 0.57 | 0.51 | 0.31 | 0.10 | 0.07 | 0.48 | 0.39 | 0.30 | 0.20 | 0.18 |
| rsd | 1.14 | 1.06 | 1.12 | 0.54 | 0.45 | 1.15 | 1.23 | 0.84 | 0.63 | 0.67 |
| sodC | 1.56 | 1.84 | 1.36 | 5.54 | 10.84 | 1.56 | 1.24 | 1.95 | 2.27 | 2.33 |
| sra | 0.98 | 1.07 | 1.27 | 2.18 | 2.06 | 1.03 | 1.15 | 1.48 | 1.01 | 1.26 |
| talA | 1.08 | 1.25 | 1.43 | 1.91 | 2.24 | 1.20 | 1.15 | 1.25 | 1.40 | 1.43 |
| tam | 0.87 | 1.02 | 3.18 | 5.63 | 8.63 | 1.42 | 2.02 | 2.52 | 2.03 | 3.19 |
| tktB | 1.03 | 1.13 | 1.28 | 1.57 | 2.01 | 1.04 | 1.08 | 1.20 | 1.16 | 1.20 |
| treA | 1.43 | 2.00 | 2.40 | 2.88 | 2.90 | 1.37 | 1.45 | 1.68 | 1.62 | 1.46 |
| uspB | 6.88 | 6.29 | 6.99 | 5.62 | 4.39 | 6.84 | 7.64 | 6.36 | 4.54 | 3.66 |
| wrbA | 1.02 | 1.14 | 1.44 | 1.84 | 2.18 | 0.97 | 1.05 | 1.25 | 1.29 | 1.39 |
| ybjP | 1.14 | 1.52 | 2.02 | 2.91 | 3.69 | 1.02 | 1.18 | 1.45 | 1.46 | 1.60 |
| yccJ | 1.30 | 1.62 | 2.31 | 4.28 | 6.36 | 1.23 | 1.51 | 1.86 | 2.03 | 2.28 |
| yciE | 1.01 | 1.07 | 1.27 | 1.46 | 2.97 | 1.08 | 1.10 | 1.41 | 1.34 | 1.09 |
| yciF | 1.47 | 1.60 | 2.54 | 5.53 | 10.13 | 1.51 | 1.55 | 1.60 | 1.33 | 0.80 |
| yciG | 4.63 | 77.65 | 6.38 | 425.50 | 525.30 | 11.18 | 11.50 | 14.59 | 8.43 | 35.21 |
| ydcS | 2.86 | 5.16 | 5.94 | 8.70 | 9.24 | 2.18 | 2.97 | 3.19 | 2.80 | 2.97 |
| ydcT | 19.04 | 29.47 | 57.32 | 77.49 | 75.01 | 11.47 | 27.76 | 31.02 | 25.81 | 26.65 |
| yegQ | 0.90 | 0.84 | 0.65 | 0.40 | 0.25 | 0.92 | 0.87 | 0.68 | 0.61 | 0.49 |
| yegS | 0.65 | 0.84 | 1.60 | 2.23 | 3.43 | 0.90 | 1.09 | 1.03 | 1.19 | 1.37 |
| ygaU | 1.60 | 1.80 | 2.56 | 3.78 | 6.22 | 1.40 | 1.65 | 1.85 | 1.75 | 1.95 |
| yhjG | 1.31 | 1.62 | 1.25 | 1.49 | 2.10 | 1.91 | 0.82 | 0.78 | 1.87 | 1.43 |
| yjcH | 7.82 | 10.70 | 17.22 | 22.24 | 24.90 | 4.15 | 7.31 | 9.49 | 6.16 | 6.72 |

Fold-change in concentrations of proteins regulated by σ^S^ in cells during the entry into persistence and starvation, compared to cells growing on glucose. Almost half of the proteins regulated by σ^S^ are more than 2-fold up- or down- regulated during the entry into persistence.

## Appendix Table S6 – Change in antibiotic tolerance to ampicillin after nutrient-shift in mutant strains.

| Strain | Fraction of antibiotic-tolerant cells in the mutant strain | Fraction of antibiotic-tolerant cells in the respective WT background | Change in fraction of the antibiotic-tolerant cells compared to the respective WT (±SD) | p-value (t-test) |
| --- | --- | --- | --- | --- |
| MG1655 Δ10 | 0.953 ± 0.016 | 0.963 ± 0.023 | -0.01 ± 0.027 | 0.26 |
| MG1655 Δ10ΔrpoS | 0.695 ± 0.033 |  | **-0.268** **± 0.04** | **0.001** |
| BW25113 Δrmf | 0.960 ± 0.016 | 0.955 ± 0.009 | +0.005 ± 0.018 | 0.6 |
| BW25113 ΔrelA | 0.954 ± 0.011 |  | -0.001 ± 0.015 | 0.88 |
| BW25113 ΔrpoS | 0.571 ± 0.069 |  | **-0.384 ± 0.07** | **<0.001** |

## Appendix Table S7 – Primers used for TAS transcript quantification

| Gene name | Classification | Forward primer | Reverse primer |
| --- | --- | --- | --- |
| *mazE* | TAS-AT | ATGATCCACAGTAGCGTAAAGCGT | ATTGAGCGCCTGCATTAACGTA |
| *chpS* | TAS-AT | TGCTGGCACAGTGTGACATG | GGGTGGATTTACCCCAGACA |
| *hicA* | TAS-T | TGGGAGGCGCAGTGTCA | TTTACGCAATGGTTCTTTAATCTCA |
| *mqsR* | TAS-T | CGCACACCACATACACGTTTG | ACTTGCCCGGCATTGACA |
| *rnlA* | TAS-T | GCTCGTTTCAGGGCTATGTGTT | AATGGTGCGTAGTTCAGGATAAAGT |
| *relB* | TAS-AT | GACGATGAACTTAAAGCGCGTT | GAAGGAGTTACACCCATTTTTTCAAG |
| *yefM* | TAS-AT | CGCTGGAAGAGACGGCTTAT | AGGCTATCGATTGAGTCCATCAA |
| *yafN* | TAS-AT | AACAAGAGGAGAAAAAGCCCATAA | GCGTGTAATTTCCTCTAATCTTGCA |
| *dinJ* | TAS-AT | AGAATCAGGCAGCGGACGTA | TGCGAACCAGGTCAGAGATG |
| *higB* | TAS-T | GCATTGAAAGATGCTGCGGA | CCAGAGCCACCAACTCCGTT |
| *ratA* | TAS-T | GTTGTACCGGAAGTCGGATTCT | TGATCCCAGCCTTAGAGACATCTA |
| *hipB* | TAS-AT | TGGGAGGCGCAGTGTCA | TTTACGCAATGGTTCTTTAATCTCA |
| *yjhX* | TAS-T | GTCAGGAACAACATACCTTACACGTT | AGTGACGCGGCCTGAAGA |
| *argE* | Housekeeping | CGATACGGTGCCATTTGATG | TCATGCTCCGTCAGTGTAAACG |
| *rhlB* | Housekeeping | CGTGGCGATCTGGATATTCTG | TGCCGGAATATGCAAACCA |
| *panC* | Housekeeping | CCTGCTTCGGTGAAAAAGATTT | GCCCATATCGGCAACCATT |
| *tufA* | Housekeeping | CACCCCGACCCGTCACTAC | CACCGGTGATCATGTTTTTAACA |
| *rplA* | Housekeeping | ACTGTACTGCCGCACGGTACT | GCGTTTGCACCTTGGGTAA |
| *gapA* | Housekeeping | GCTCAGAAACGTTCTGACATCGA | CAGCATGTATGCCATGTAATCAG |

## Appendix Table S8 – Settings of STEM software

| Section | Setting | Value |
| --- | --- | --- |
| Main Window | No normalization/add 0 | checked |
| Main window | Gene Annotation Source | Escherichia coli (EcoCyc & EcoliHub) |
| Main window | Clustering Method | STEM Clustering Method |
| Main window | Maximum Number of Model Profiles | 30 |
| Main window | Maximum unit Change in Model Profiles between Time Points | 2 |
| Filtering | Maximum Number of Missing Values | 0 |
| Filtering | Minimum Correlation between Repeats: | 0 |
| Filtering | Minimum Absolute Expression Change | 1 |
| Filtering | Change should be based on | Difference from 0 |
| Filtering | Pre-filtered Gene File | <empty> |
| Model Profiles | Maximum Correlation | 1 |
| Model Profiles | Maximum Number of Candidate Model Profiles | 1000000 |
| Model Profiles | Number of Permutations per Gene | 0 (all permutations) |
| Model Profiles | Significance Level | 0.05 |
| Model Profiles | Permutation Test Should Permute Time Point 0 | Unchecked |
| Model Profiles | Correction Method | False Discovery Rate |
| Clustering Profiles | Minimum Correlation | 0.6 |
| Clustering Profiles | Minimum Correlation Percentile (repeat only) | 0 |

Unspecified settings were left at default values. Short Time-series Expression Miner (STEM) version 1.3.8 was used for analysis.

# Appendix Texts

## Appendix Text S1: Evaluation of the effects of the presence of non-persister cells on the determined persister phenotype

In this text, we demonstrate that the small fraction of growing cells occurring after a glucose to fumarate shift does not significantly influence the reported values describing the phenotype of persister cells. For this analysis, we used two datasets: (i) a dataset describing cells growing normally on fumarate and (ii) a dataset describing the mixed population obtained after a glucose to fumarate shift (i.e. containing mostly persisters, and some cells growing on fumarate). We also used the information about the actual fractions of cells growing on fumarate in this mixed population. These datasets allowed us to determine the effect that the cells growing normally on fumarate would have on the reported measurements from the mixed population.

### Worst-case scenario

After a switch from M9-glucose to M9-fumarate medium a small fraction of cells adapt to the new growth medium, while the rest adopt a non-/slow-growing, antibiotic-tolerant phenotype (Kotte et al., 2014). In the current paper, we confirm this finding in Appendix Figure S1A. From the cell population used to inoculate the new growth medium, only 0.1 ± 0.05% (SD) of cells adapt to grow on fumarate. 10-15 hours after the switch, the growing population reaches 1% of total population (Appendix Figure S1A). Because of the inherent difficulty in measuring small fractions of cells, we can only estimate how many growing cells are present at 8 hours after the switch, which is the time point until which we characterized the persister cells. To do a worst-case estimation of the effect of growing cells on the determined persister phenotype, we assumed that 1% of normally growing cells is already reached 8 hours after the switch. For the estimation of the effect of the presence of the growing cells, we further assumed that the growing cells have already reached their new steady state 8 hours after the switch (which is also a conservative estimate).

### Formula for calculating the effect of growing cells on results of persister cells

If our population is a mixture of two populations, every quantified value will be result of a weighted average. In our particular case, each value can be expressed as *X = aY + bZ*, where *Y* is the true value for persister cells and *Z* is the true value for growing cells, *a* is the fraction of persister cells and *b* is the fraction of growing cells. Consequently, we can calculate the true value for slow-growing cells: *Y = ( X – bZ ) / a*. We will use this above formula in the following to show that the influence of growing population does not elicit a statistically significant difference. We propagated errors according to *ΔY = √(ΔX^2^+(bΔZ)^2^))/a*.

### Growing population does not influence the physiological data

Physiological rates of persisters were de-convoluted using the formulas described above, the measured physiological rates of mixed populations and the measured physiological rates of fumarate-adapted cells (Appendix Figures S1B and S1C). Here, we found that the 95% confidence interval of the mean values of calculated rates overlap between the mixed-population and de-convoluted values in the worst-case scenario.

### Growing population does not influence the metabolite concentration measurements

Metabolite concentrations were de-convoluted as described above. For all measured metabolites, the 95% confidence interval of the mean values of metabolite concentrations overlapped between concentrations we report and the de-convoluted concentration (Appendix Figure S1D). Thus, the possible difference elicited by growing cells in this worst-case-scenario is smaller than the accuracy of the method.

### Growing population does not influence the protein concentration measurements

Also for the proteome data, we applied the same deconvolution method. Then, we compared correlation coefficients of the mixed population proteome and the de-convoluted proteome to detect differences between proteomes. We found that their Pearson’s r equals 0.99998. The average Pearson’s r between three biological replicate measurements of glucose-growing cells’ proteomes is equal to 0.989. Thus, the possible difference elicited by the small fraction of growing cells even in the worst-case-scenario is smaller than the accuracy of the method and thus negligible.

### Concluding remarks

Together, even considering a worst-case-scenario (assuming that at 8 hours already 1% of cells belong to the growing phenotype), the small fraction of growing cells does not affect the values that we report for the persister cells in a significant manner.

# Appendix Reference List

Keseler, I.M., Mackie, A., Peralta-Gil, M., Santos-Zavaleta, A., Gama-Castro, S., Bonavides-Martinez, C., Fulcher, C., Huerta, A.M., Kothari, A., Krummenacker, M.*, et al.* (2013). EcoCyc: fusing model organism databases with systems biology. Nucleic Acids Res. *41,* D605-D612.

Kotte, O., Volkmer, B., Radzikowski, J.L., and Heinemann, M. (2014). Phenotypic bistability in Escherichia coli's central carbon metabolism. Molecular Systems Biology *10,* 736.
